# The Role of Aryl-Hydrocarbon Receptor (AhR) in Osteoclast Differentiation and Function

**DOI:** 10.3390/cells9102294

**Published:** 2020-10-14

**Authors:** Robin Park, Shreya Madhavaram, Jong Dae Ji

**Affiliations:** 1 MetroWest Medical Center/Tufts University School of Medicine, Framingham, MA 01702, USA; robin.park@mwmc.com (R.P.); shreya.madhavaram@mwmc.com (S.M.); 2 Department of Rheumatology, College of Medicine, Korea University, Seoul 02841, Korea

**Keywords:** aryl-hydrocarbon receptor, osteoclastogenesis, bone remodeling, cytochrome P450

## Abstract

Aryl hydrocarbon receptor (AhR) is a ligand-activated transcription factor that plays a crucial role in bone remodeling through altering the interplay between bone-forming osteoblasts and bone-resorbing osteoclasts. While effects of AhR signaling in osteoblasts are well understood, the role and mechanism of AhR signaling in regulating osteoclastogenesis is not widely understood. AhR, when binding with exogenous ligands (environmental pollutants such as polycylic aryl hydrocarbon (PAH), dioxins) or endogenous ligand indoxyl-sulfate (IS), has dual functions that are mediated by the nature of the binding ligand, binding time, and specific pathways of distinct ligands. In this review, AhR is discussed with a focus on (i) the role of AhR in osteoclast differentiation and function and (ii) the mechanisms of AhR signaling in inhibiting or promoting osteoclastogenesis. These findings facilitate an understanding of the role of AhR in the functional regulation of osteoclasts and in osteoclast-induced bone destructive conditions such as rheumatoid arthritis and cancer.

## 1. Introduction 

Since the role of AhR in immune cells has been extensively summarized in previous review articles, we will briefly describe the major functions of AhR in key immune cells here [2,3,4]. The immunomodulatory effects of AhR occur primarily through effector T cells and regulatory T cells (Treg) via direct and indirect mechanisms such as modulation of antigen-presenting-cell (APC) activity. AhR activation leads to an increase in FoxP3+ Treg cells via various mechanisms, including modulation of FoxP3 expression and direct transactivation [5,6]. Furthermore, AhR activation appears to influence the differentiation of a subset of precursor Th17 cells into either a regulatory T cell or a fully Th17 cell phenotype [7]. More importantly, in conventional dendritic cells (DC), the consequences of AhR activation are more nuanced with ligand-dependent effects. For example, in the context of antigen presentation, the AhR agonist 2,3,7,8-Tetrachlorodibenzo-p-dioxin (TCDD) decreases CD11c expression and increases MHC-II, CD86, IL-6, and TNF-κ in murine DCs [8,9]. On the other hand, when murine DCs were treated with the AhR agonist 2-(1′H-indole-3′-carbonyl)-thiazole-4-carboxylic acid methyl ester (ITE), MHC-II and costimulatory molecules as well as Th1 and Th17 polarizing cytokine production were decreased [10]. Furthermore, AhR activation inhibits the differentiation of human monocytes into Langerhans DCs in vitro, while AhR inhibition via StemRegenin 1 induces human CD34+ stem cell progenitor differentiation into mature DCs [11]. Therefore, AhR activation has context-dependent effects on the function and differentiation of DCs. 

Aryl-hydrocarbon receptor (AhR) is a ligand-activated transcription factor of the Pern-Arnt-Sim (PAS) superfamily initially implicated in xenobiotic metabolism of environmental pollutants; however, recent studies have also elucidated the role of AhR in immune regulation and bone remodeling [1].

Tobacco smoke, which is a well-established risk factor in bone-remodeling disorders, including osteoporosis, contains numerous environmental toxins, including polycyclic aryl hydrocarbons (PAH) such as benzo[a]pyrene (BaP) and dioxins such as TCDD; importantly, both BaP and TCDD are well-established AhR agonists [12].

**Figure 1:**
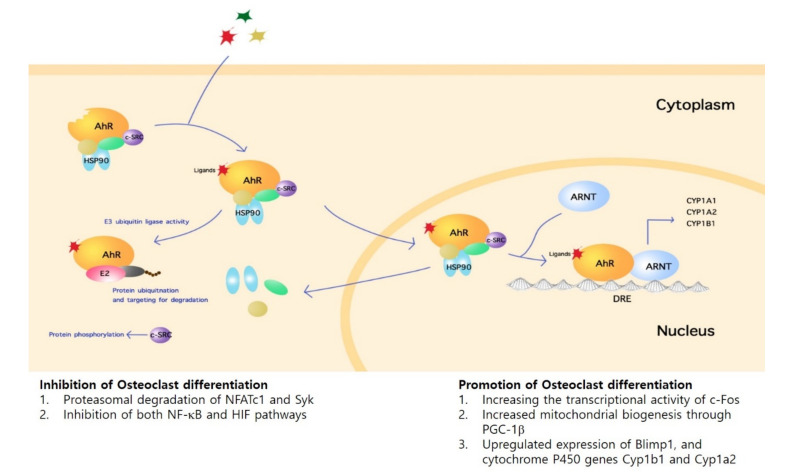
Aryl-hydrocarbon Receptor (AhR) Signaling Pathway in Osteoclasts. AhR is localized in the cytoplasm in multiprotein complex including c-src and HSP90. Interaction with an agonist results in translocation of the complex to the nucleus. In complex with AhR nuclear translocator (ARNT), AhR controls gene transcription by binding dioxin-response elements (DRE)/ xenobiotic-responsive elements (XRE) containing genes. AhR signaling includes non-genomic pathways such as the release of c-src kinase with consequent target protein phosphorylation and AhR functioning as an E3 ubiquitin ligase. Such intracellular signaling mechanisms lead to either the activation or inhibition of osteoclast differentiation and function.

Following activation of AhR, intracellular signaling occurs via either the genomic or non-genomic pathway. At steady state, AhR remains in a multi-protein complex that include heat shock protein 90 (HSP90) and c-src protein kinase localized in the cytoplasm. Upon ligand-binding, AhR undergoes nuclear translocation, forms a complex with AhR nuclear translocator (ARNT), then exerts transcriptional control over target genes harboring dioxin-response elements (DRE), or xenobiotic-responsive elements (XRE) such as the cytochrome P450 family 1 subfamily A member 1 (Cyp1a1) and cytochrome P450 family 1 subfamily B member 1 (Cyp1b1) [13]. On the other hand, non-genomic AhR signaling pathways may also lead to gene expression regulation. For instance, the release of c-src from the multi-protein complex upon ligand binding leads to phosphorylation of target proteins [14]. Furthermore, in addition to its function as a transcription factor, AhR also acts as an E3 ubiquitin ligase that leads to ubiquitination and targeting for degradation in the proteasome [15] (Figure 1).

Homeostasis in bone remodeling depends on relative levels of bone formation and resorption, which in turn depend primarily on the relative activities of osteoblasts (OB) and osteoclasts (OC). In this context, AhR signaling leads to modulation of the NF-κB, Wnt, and MAP kinase pathways; influences the function and differentiation of OB and OC; and results in altered bone remodeling [16,17,18,19,20]. The overall effect of AhR activation in OB is suppressed cell differentiation. First, AhR activation by TCDD inhibits the differentiation of bone-marrow-derived stem cells into OB [21]. In in vivo models, TCDD leads to bones with thin cortices, a harder matrix, and a greater trabecular fraction, resulting in overall mechanical weakness [22]. Second, AhR inhibition by resveratrol leads to an increase in bone mineral density (BMD) and bone strength in murine models [23]. Overall, AhR agonism has dose-dependent effects on OB in which hyperactivation and hypoactivation, respectively, inhibit and promote bone formation. 

Indeed, recent studies provide a relatively unified description of the effects of AhR signaling in OB; however, the effects of AhR on OC function and differentiation remain unclear. Therefore, the aim of this review is to summarize the pertinent published findings on AhR signaling in OC, provide a consistent view of the effects of AhR activation on OC function and differentiation, and provide a more complete description of the role of AhR signaling in bone remodeling in health and disease.

## 2. Role of AhR in Osteoclast Differentiation and Function 

AhR is well-known to be expressed in bone cells, including OB and OC [12,24]. The activation of AhR by a representative uremic toxin and putative endogenous ligand indoxyl-sulfate (IS) affects osteoclastogenesis in a dose-dependent, exposure duration-dependent manner. Low doses of IS with short exposure duration (3 days) stimulated differentiation of osteoclast precursor cells. On the other hand, longer exposures (5 days) led to suppressed osteoclast differentiation [25]. Thus, dose and duration of agonist exposure are important variables in AhR-mediated modulation of OC differentiation by IS.

Furthermore, the effects of AhR activation by PAH are also dependent on agonist dose as well as cell density. BaP is pro-osteoclastogenic in vitro when cell density is low and anti-osteoclastogenic when cell density is high. At low concentrations, BaP is highly pro-osteoclastogenic, whereas at high concentrations (2 μM) its effects on osteoclasts are nonexistent in vitro [26]. Consistent with these findings, BaP at high concentrations (10^−5^ M) reduces osteoclastogenesis in the cell line RAW264.7 [27]. Moreover, in addition to the TCDD-mediated dose-dependent suppression of osteoclastogenesis, short durations of exposure to TCDD do not alter the function of OC, suggesting that duration of exposure is an important variable in PAH-mediated AhR modulation [12,28]. Thus, OC differentiation and function are modulated by PAH through AhR in a dose-dependent, cell-density-dependent, and duration-dependent manner.

AhR appears to also be required for basal receptor activator of nuclear factor kappa beta ligand (RANKL)-induced osteoclastogenesis. Osteoclastogenesis is suppressed in AhR null cell cultures, unlike its AhR proficient counterpart. These findings are recapitulated in vivo where mice genetically deficient in AhR or the downstream effector genes, Cyp1a1 or Cyp1b1, demonstrate reduced bone resorption and high bone mass. Consistently, BaP-mediated AhR activation in wild-type mice stimulates osteoclastogenesis and osteoclast function [26]. In addition to mediating the proosteoclastogenic effects of BaP, AhR itself contributes at least in part to RANKL-induced osteoclastogenesis. Cyp1 gene expression is modulated by AhR as well as RANKL. RANKL strongly induces Cyp1a1 and Cyp1a2 expression in wild-type cells, but not in AhR null cells. Bone marrow cell cultures from Cyp1a1/1a2^−/−^ double knockout and Cyp1a1/1a2/1b1^−/−^ triple knockout mice demonstrate strongly suppressed osteoclastogenesis, albeit less marked than AhR^−/−^ mice. Furthermore, TCDD administration is unable to stimulate osteoclastogenesis in cell cultures from Cyp1a1/1a2^−/−^ double knockout and Cyp1a1/1a2/1b1^−/−^ triple knockout mice, unlike in cell cultures from wild-type mice. These results are recapitulated via Cyp1 inhibitors such as proadifen and tetramethylsilane (TMS), which suppress osteoclastogenesis in dose-dependent fashion [26]. Taken together, these findings suggest that AhR-induced Cyp1 expression mediates at least in part the induction of osteoclastogenesis by BaP and RANKL. 

In addition, in transgenic mice engineered to express constitutively active AhR (CA-AhR), total osteoclast resorptive activity is heightened in female mice but not in males. On the other hand, the indirect index for total number of OC in the entire skeleton remains unchanged and the RANKL/osteoprotegerin (OPG) is higher in female CA-AhR mice. Furthermore, there is increased osteoclast volume and serum levels of c-terminal telopeptide (CTX) and cathepsin K, suggesting augmented trabecular bone resorption in female CA-AhR mice [29]. The reason for the sex-specific effects of dioxin AhR agonists such as TCDD as noted above depends on endogenous estrogen status. Supporting this finding is that in ovariectomized mice that demonstrate an estrogen-deficient phenotype, TCDD induces an estrogenic response in certain tissue types such as the uterus; in contrast, AhR agonists have estrogen antagonist effects in presence of estrogen [30,31]. In addition, these gender-specific effects in osteoclastogenesis are not limited to AhR function. For example, osteoclastogenesis in the presence of M-CSF and RANKL differs between BMM derived from male versus female mice [32]. Taken together, results indicate that AhR activation has gender-specific, in particular estrogen-specific, OC-augmenting effects. 

The estrogen receptor (ER) ligands estradiol (E2), 4-hydroxy-TAM (4-OHT), and raloxifene suppress OC differentiation. Moreover, the suppressive effects of 4-OHT on OC differentiation were nullified via cotreatment with the AhR antagonist, α-naphthoflavone (ANF). Thus, the inhibitory effects of 4-OHT on OC differentiation are mediated at least in part by AhR. On the other hand, neither the synthetic ER ligand ICI 182,780 (ICI) nor raloxifene are significantly capable of activating AHR in OCs despite their ability to induce robust Cyp1a1 expression in breast cancer cells, suggesting that AhR agonist activity is both cell-type and agonist-type dependent [33]. 

TCDD is associated with dose-dependent suppression of bone growth in animal models at doses relevant to environmental exposure and health risk [34,35]. OC differentiation is mechanistically inseparable from OB differentiation, as OB produces key regulators of OC differentiation such as RANKL and M-CSF [36,37]. TCDD as well as 3-MC suppress OB differentiation; however, whereas TCDD does not affect mature OB despite strong AhR expression, 3-MC downregulates the expression of RANKL, which is required for OC differentiation [12,38,39]. In addition, BaP directly inhibits osteoclast formation and function, where this inhibitory activity is dependent on AhR-RANKL crosstalk [28]. 

In the setting of co-culture of osteoclast precursor cells and bone marrow stromal cells, the PAH AhR agonists BaP and 7,12-dimethylbenz-[a]anthracene (DMBA) decrease the number of OC independently of agonist dose at high cell densities. In contrast, in the setting of RAW 264.7 cells cultured with RANKL, BaP and DMBA increased OC numbers at low cell densities (<100 TRAP+ cells/well). These findings suggest the possibility of stromal cells, OC, or OC precursors determining the effects of BaP and DMBA on osteoclastogenesis. Furthermore, resveratrol as well as high concentrations of RANKL reverses BaP-induced inhibition of OC function. The expression of Cyp1b1 is strongly induced by BaP (10^−5^ M) in OC cultures. While this BaP-mediated effect remained unaffected by low concentrations of RANKL (25 ng/mL), high concentrations (200 ng/mL) were able to reverse the effects [27].

Taken together, AhR agonists modulate cell differentiation and function with the direction of overall effect depending on agonist dose, treatment duration, and cell density in OC. Furthermore, AhR is implicated in RANKL-induced osteoclastogenesis, wherein RANKL induces the expression of the AhR-related genes, Cyp1a1 and Cyp1a2. In turn, the pro-osteoclastogenic actions of RANKL and AhR agonists such as BaP are mediated by AhR-induced Cyp1 expression. The results of studies demonstrating the effects of specific AhR ligands on osteoclast differentiation and function have been summarized elsewhere (Table 1).

## 3. Regulatory Mechanisms of AhR in Osteoclasts

Understanding the mechanistic basis of signaling pathways through which AhR regulates osteoclast differentiation and therefore bone remodeling has significant implications for bone disease, particularly in the role of environmental pollutants in inducing bone loss. Increasing evidence suggests that AhR regulates osteoclastogenesis through inhibition and promotion in ligand-, species- and concentration-specific manners.

### 3.1. Inhibitory Mechanisms

#### 3.1.1. Direct Mechanisms

AhR can inhibit osteocleogenesis through several different pathways. One important pathway is through AhR functioning as a ligand-dependent E3 ubiquitin ligase that induces the proteasomal degradation and ubiquitination of osteoclastogenesis promoting target proteins [12]. When Raw 264.7 cells with sRANKL were cultured with high concentrations of Indoxyl Sulfate (>500 μM), a protein binding uremic toxin common in CKD, there was decreased ARNT expression along with increased NFATc1 ubiquitination. This correlation suggested that ARNT served as a molecular switch with decreased levels disabling AhR ligand-activated transcriptional activity, leading to AhR functioning as an E3 ubiquitin ligase, increasing proteasomic degradation of NFATc1, and therefore inhibiting osteoclast precursor differentiation [25]. Another toxin (tetrandine), a natural alkaloid, along with 3,3′-diindolylmetheane (DIM), facilitate AhR-mediated ubiquitiation through AhR-c-src-c-Cbl pathway. Tetrandine and DIM phosphorylate protein tyrosine kinase c-src, which then dissociates from Ahr-c-src complex and induces AhR-mediated activation of E3 ubiquitin ligase c-CBL and subsequent ubiquitination and degradation of Syk, inhibiting osteoclastogenesis (through inhibition of NFATc1) and bone destruction in arthritis. This was demonstrated through oral administration of tetrandrine in collagen-induced arthritis rats, which decreased the number of phospho-Syk-positive cells and osteoclasts and resulted in reduced bone erosion in the areas of the proximal tibial epiphysis excluding the cortical bone [41]. While IS-mediated ubiquitination and degradation of NFATc1 occurs through interplay with ARNT, tetrandrine and DIM did not change in the absence of ARNT, which suggests that they activated a non-genomic route of AhR ubiquitation and degradation.

Another mechanism of PAH-induced inhibition of osteoclastogenesis is through inhibition of essential components of RANKL-induced signaling. BaP, a typical PAH present in cigarette smoke, inhibits osteoclastogenesis through crosstalk between RANKL and AhR competing for NF-κB, a common transcription factor in both pathways. BaP was found to inhibit RANKL-induced NF-κB activation and nuclear translocation at early time points and induce smaller-amplitude, sustained activation of NF-κB at later time points through activation of AhR (6–24 h). NF-κB involvement in the BaP-mediated signaling pathway was confirmed with incubation of two different NF-κB inhibitors, which resulted in a dose-dependent decrease of BaP-induced Cyp1b1 gene expression. Co-immunoprecipitation demonstrated that AhR interacted with NF-κB p65 in RAW264.7 cells and BaP enhanced this interaction. However, in the presence of high concentrations of RANKL, there was no interaction observed between AhR and p65 due to sequestration of NF-κB to RANKL-specific kB elements [44]. In a similar manner, norisoboldine, an isoquinoline alkaloid, induces anti-osteoclastogenesis activity through attenuating RANKL-induced OC differentiation through AhR-dependent inhibition of NF-κB-p65 nuclear translocation. Normally, RANKL stimulation markedly decreases the AhR-NF-κB-p65 complex, whereas treatment with norisobordine (NOR) markedly augmented this complex through possible agonism of the AhR receptor. Furthermore, NOR was found to inhibit RANKL-induced activation of HIF-1κ signaling pathway, an important pathway in regulating osteoclast mediated bone resorption, through AhR. NOR increases AhR activation to partner molecule ARNT, preventing it from dimerizing with HIF-1κ, therefore inhibiting expression of downstream genes such as VEGF and consequent differentiation of OC. NOR was also independently found to up-regulate Cyp1a1 expression.

#### 3.1.2. Indirect Mechanisms

While the inhibitory effects of PAHs on osteoclasts can be direct such as through the ubiquitination of syk and NFATc1 or through the competitive interplay of AhR and NF-κB, they can also be indirect such as inhibiting RANKL via the stromal cell population. 3-Methylcholanthrene (3MC), an aromatic hydrocarbon that binds to AhR, dose-dependently inhibited the formation of mono- and multinuclear osteoclast-like cells through the inhibition of RANKL expression in osteoblast-supporting stromal cells, ST2 cells. This could be through the mechanism of 3MC inhibiting expression of mRNA for RANKL resulting in lower RANKL levels but not affecting that for OPG (decoy receptor for RANKL) or macrophage colony stimulating factor (M-CSF). Furthermore, 3MC did not inhibit the formation of osteoclast-like cells from mouse spleen cells when supported by the exogenous soluble RANKL and M-CSF in a dose-dependent manner. This illustrates that inhibition of OC could be due to 3MC-induced alteration of RANKL expression in ST2 cells [45]. It is important to note that this is different from other studies, as 3MC is an indirect inhibitor of osteoclastogenesis by acting on stromal cells.

### 3.2. Promotion Mechanisms

While AhR plays a critical role in several key signaling pathways in inhibiting osteoclastogenesis, it also plays an equally pivotal role in promoting osteoclast formation and differentiation. This is done through a couple key regulatory mechanisms. For example, through utilization of AhR knockout mice, it was found that regulation of bone mass may be mediated by expression of AhR target genes, including B lymphocyte-induced maturation protein (Blimp1), along with cytochrome P450 genes Cyp1b1 and Cyp1a2 expression. These transcriptional target genes play an essential role in osteoclast formation and bone resorption. More specifically, Blimp1 functions as a transcriptional repressor of anti-osteoclastogenic genes such as *Irf8*, *MAfB*, and *Bcl6* [49]. Functional regulation of AhR could also be linked to the suppression of *Stat5b* signaling in osteoclasts [42]. The significance of AhR was further solidified through comparison of an AhR agonist in control mouse and in knockout mice. When control mice were treated with 3MC, an AhR agonist, they exhibited decreased bone mass and increased bone resorption, which were not observed when 3MC was administered to knockout mice with AhR deletion [42].

Increased osteoclast differentiation was also found to be influenced through RANKL-dependent expression of AhR and upregulation of c-Fos. Bone marrow macrophages (BMMs) were treated with RANKL, which increased expression level of AhR. In BMM cells deficient in AhR (AhR^−/−^ cells), RANKL-stimulated osteoclastogenic signals such as phosphorylation of Akt, MAPK, and NF-κB were impaired whereas their response to M-CSF was not affected. Furthermore, expression of AhR in BMM osteoclasts was upregulated by RANKL at an earlier stage than the expression of signature osteoclast genes such as those encoding cathepsin K and NFATc1. When BaP was administered, it induced higher levels of c-Fos in RANKL-stimulated BMMs, although c-Fos expression was not induced by BaP or RANKL in AhR^−/−^ BMMs. Furthermore, the formation of an AhR-c-Fos complex in RANKL-stimulated BMMs was required for osteoclast differentiation. Absence of AhR was also shown to diminish basal mitochondrial biogenesis in osteoclasts through lack of induction of PGC-1β mRNA and protein. These findings suggest that RANKL/AhR/c-Fos signaling axis and upregulation of PGC-1β by AhR play a critical role in the earlier stages of osteoclastogenesis [41].

AhR also plays a mechanistic role in osteoclast promotion through NF-κB augmentation. This is seen in its role in autoimmune arthritis, such as in rheumatoid arthritis. In RA, the strongest genetic risk factor is conferred through a five-amino acid sequence motif called the shared epitope (SE), which is encoded by HLA-DRB1 alleles. Transgenic mice carrying human SE-coding alleles, when exposed to AhR agonists, showed a robust increase in arthritis severity, bone destruction, overabundance of osteoclasts, and IL17-expressing cells in the inflamed joints and draining lymph nodes of arthritic mice. This mechanism is facilitated through upregulation of transcriptional activity by NF-*κ*B, which plays a key role in the SE-AhR interaction. Administration of an NF-*κ*B inhibitor blocked AhR-SE synergism in a dose-dependent fashion. These findings illustrate that SE ligand and AhR agonists operate synergistically through cross-talk by NF-*κ*B and significantly enhance OC and Th17 cell differentiation, playing a significant role in precipitating inflammation and bone destruction in autoimmune arthritis [47].

## 4. Conclusions

Osteoclasts are multinucleated bone cells that are formed by fusion of mononuclear precursor cells in the presence of osteoclastogenic cytokines, including RANKL and M-CSF. RANKL binds to RANK to activate NF-κB and NFAT1c, which stimulate osteoclast differentiation. AhR, an intracellular ligand-activating transcription factor, is activated by various exogenous and endogenous ligands and it turn activates various signaling pathways that interplay with RANKL-RANK pathway and play a crucial role in modulating osteoclast differentiation and function. Among these pathways, AhR is essential in mediating RANKL-stimulated expression of c-Fos, suggesting that the Ahr-c-cFos pathway is important in Ahr-mediated osteoclast stimulation.

Understanding this mechanistic basis of AhR-AhR ligand signaling pathways in osteoclastogensis and the subsequent disruption of bone remodeling is important for understanding factors that contribute to pathological bone remodeling. Overall, the aim of this review is to provide insight into the role of AhR agonists in osteoclast differentiation. The inhibition/promotion mechanisms of osteoclastogenesis through AhR agonists and various signaling pathways is explored in a ligand-, duration-, and concentration-specific manner. Lastly, there is a paucity of research conducted in human OC in the context of AhR modulation. Notably, there are marked differences in the regulation of osteoclastogenesis in mice and humans. For instance, IL-27 acts as a potent direct inhibitor of human osteoclast precursor cells, whereas in mice, IL-27 mainly acts as an indirect modulator of osteoclast differentiation through cells other than osteoclast precursors. Furthermore, in humans, triggering receptor expressed on myeloid cells 2 (TREM-2) deficiency is associated with impaired osteoclast differentiation and bone remodeling as well as Nasu–Hakola disease whereas in mice, deficiency in TREM-2 has unclear effects in vivo and has osteoclastogenic effects in vitro. Therefore, further research on the effects and mechanisms of AhR activation in OC is warranted in human OCs. Elucidation of AhR function in human osteoclasts will introduce a potential therapeutic target for various human diseases in which osteoclasts are implicated in pathogenesis, including bone destructive diseases such as osteoporosis and cancer.

## Figures and Tables

**Table 1:** Summary of studies on effects and mechanisms of AhR ligands in vitro and in vivo.

Study	Experimental Model	Agonist	Antagonist	Effect on Osteoclastogenesis	Mechanism of Action
In vitro studies					
Koskela 2012 [40]	BM cells	TCDD	N/A	Inhibition	N/A
Izawa 2016 [41]	BM cells	BaP	N/A	Promotion	Via c-Fos–NFATc1 and mitochondrial biogenesis through PGC-1β
Yu 2015 [42]	BM cells	3-MC	N/A	Promotion	Blimp1, Cyp1b1, and Cyp1a2 expression was downregulated in the absence of AhR
Voronov 2005 [27]	RAW264.7 cells	BaP	Resveratrol	Inhibition	N/A
Liu 2020 [25]	RAW 264.7 cells	Indoxyl-sulfate	CH223191 or siRNA	Agonist dose and duration dependent: -Promotion (short-term, low-dose) -Inhibition (long-term, high-dose)	Different IS levels switch the role of AhR from that of a ligand-activated transcription factor to that of an E3 ubiquitin ligase
DuSell 2010 [33]	RAW264.7 cells	4-OHT (4-hydroxy-TAM)	α-naphthoflavone siRNA	Inhibition	N/A
Wei 2015 [43]	RAW 264.7 cells	Norisoboldine (NOR)	Resveratrol α-naphthoflavone	Inhibition	Inhibition of both NF-κB and HIF pathways
Voronov 2008 [44]	RAW264.7 cells	BaP	N/A	Inhibition	Consequence of crosstalk between AhR and RANKL signaling pathways competing for the common transcription factor NF-kB
Naruse 2004 [45]	Mouse spleen cells and clonal osteogenic stromal ST2 cells	3-MC	N/A	Inhibition	Via the inhibition of RANKL expression in osteoblastic cells
Korkalainen 2009 [28]	Haematopoietic stem cells	TCDD	N/A	Inhibition	N/A
Ilvesaro 2009 [12]	Rat osteoclasts from long bones	TCDD	N/A	No effect	N/A
Iqbal 2013 [26]	In vitro: BM cells, RAW-C3 cells In vivo: mice with BaP, TCDD oral gavage	BaP, TCDD	N/A	Promotion	N/A
Jia 2019 [46]	In vitro: BM cells RAW264.7 cells In vivo: CIA rats	Tetrandrine, DIM	CH223191 or siRNA	Inhibition	Enhanced ubiquitination and degradation of Syk through the AhR/c-src/c-Cbl signaling pathway
Fu 2018 [47]	In vitro: BM cells In vivo: SE transgenic mice	6-formylindolo[3,2-b]carbazole (FICZ), TCDD	N/A	Promotion	Interaction between SE and AhR agonists during osteoclastogenesis is mediated by the NF-κB signaling pathway
In vivo studies					
Csanaky 2018 [48]	Juvenile mice with oral gavage of TCDD	TCDD	N/A	Inhibition	N/A
Yu 2014 [23]	AhR(ΔOc/ΔOc) mice	3-MC	N/A	Promotion	N/A
Wejheden 2010 [29]	CA-AhR mice	N/A	N/A	Promotion	N/A

BaP (benzo[a]pyrene) CA-AhR (constitutively active AhR) DIM (3, 3’-diindolylmetheane) 3-MC (3-methylcholanthrene) TCDD (2,3,7,8-Tetrachlorodibenzo-p-dioxin) BM cells (bone marrow derived cells).
